# Importance of the Q/N-rich segment for protein stability of endogenous mouse TDP-43

**DOI:** 10.1038/s41598-022-19153-0

**Published:** 2022-09-02

**Authors:** Toshiya Sato, Kanako Oda, Seiko Sakai, Rika Kato, Saori Yamamori, Makoto Itakura, Yoshio Kodera, Masatoyo Nishizawa, Toshikuni Sasaoka, Osamu Onodera, Minesuke Yokoyama

**Affiliations:** 1 Department of Laboratory Animal Science, Kitasato University School of Medicine, Sagamihara, 252-0374 Japan; 2 Center for Genetic Studies of Integrated Biological Functions, Kitasato University School of Medicine, Sagamihara, 252-0374 Japan; 3 Department of Comparative and Experimental Medicine, Brain Research Institute, Niigata University, Niigata, 951-8585 Japan; 4 Department of Biochemistry, Kitasato University School of Medicine, Sagamihara, 252-0374 Japan; 5 Department of Physics, Kitasato University School of Science, Sagamihara, 252-0373 Japan; 6 Department of Neurology, Brain Research Institute, Niigata University, Niigata, 951-8585 Japan; 7 Department of Nursing, Niigata University of Health and Welfare, Niigata, 950-3198 Japan; 8 Central Institute for Experimental Animals, Kawasaki, 210-0821 Japan

**Keywords:** Amyotrophic lateral sclerosis, Animal disease models, Neurological models

## Abstract

TAR DNA-binding protein 43 kDa (TDP-43), a nuclear protein, plays an important role in the molecular pathogenesis of amyotrophic lateral sclerosis (ALS). The long-disordered C-terminal region (CTR) of TDP-43 is known to be aggregation-prone and a hotspot for ALS mutations, so elucidation of the physiological function of CTR will provide insights into the pathogenesis of ALS. The CTR has two Gly, aromatic, and Ser-rich (GaroS) segments and an amyloidogenic core divided into a hydrophobic patch (HP) and a Gln/Asn (Q/N)-rich segment. Although TDP-43 lacking the CTR is known to be unstable, as observed in knock-in mice, it is unclear which of these segments contributes to the stability of TDP-43. Here, we generated 12 mouse lines lacking the various sub-regions of CTR by genome editing and compared the embryonic lethality of homozygotes, and protein and mRNA expression levels of TDP-43. We demonstrated the functional diversity of the four segments of CTR, finding that the presence of the Q/N-rich segment greatly restored the protein stability of TDP-43. In addition, we found that the second GaroS deletion did not affect protein stability and mouse development.

## Introduction

TAR DNA-binding protein 43 kDa (TDP-43), a member of the heterogeneous nuclear ribonucleoprotein family, has emerged as a key player in the molecular pathogenesis of amyotrophic lateral sclerosis (ALS)^1,2^. TDP-43 mainly localizes in the nucleus and is involved in RNA metabolism, including splicing, that promotes cystic fibrosis transmembrane conductance regulator (CFTR) exon 9 skipping^3^. Moreover, TDP-43 is essential for mouse development^4–6^. In ALS, cytoplasmic aggregation of TDP-43 is a pathological feature that is often accompanied by nuclear loss of TDP-43^7,8^. Because the amount of nuclear TDP-43 is strictly regulated by binding of TDP-43 to the 3′-untranslated region of its own mRNA, thereby forming an autoregulatory negative feedback loop^9–13^, nuclear loss of TDP-43 can induce the upregulation of TDP-43 mRNA and produce a vicious cycle that leads to the perturbation of TDP-43 homeostasis^14,15^. This imbalance of aggregation-prone TDP-43 plays a role in the pathological mechanism of ALS.

**Figure 1:**
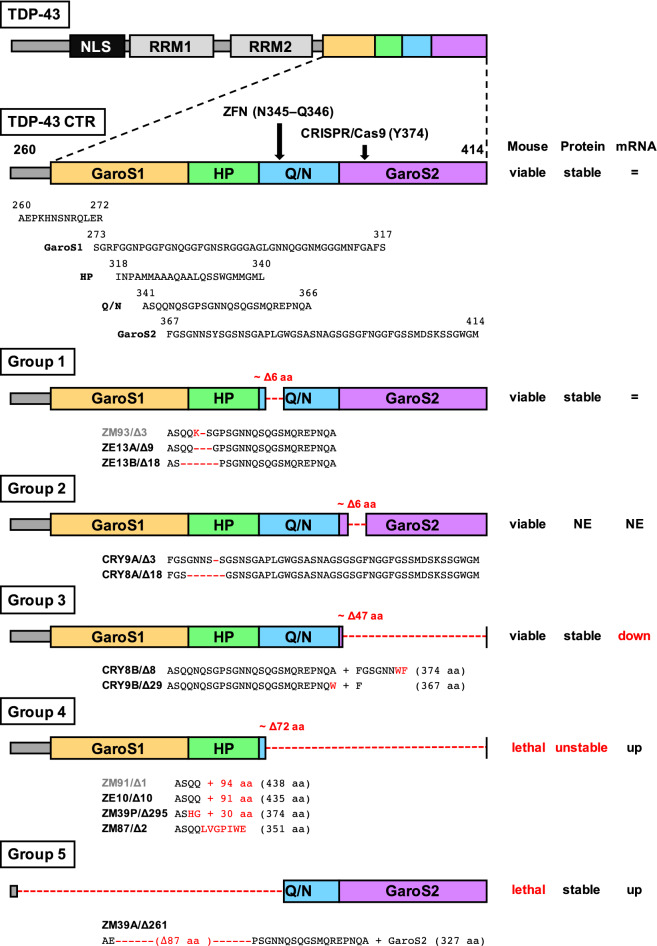
Schematic diagram of mouse TDP-43 and putative protein structures with various CTR deletions. TDP-43 contains 414 aa residues and consists of a bipartite nuclear localization signal (NLS), two RNA recognition motifs (RRM), and a long-disordered CTR. CTR is divided into four sub-regions, the GaroS1 (yellow), HP (green), Q/N-rich (blue), and GaroS2 segments (purple); the aa sequences are depicted by one-letter codes according to the TDP-43 CTR. The genome-edited mice were categorized into five groups based on the putative CTR structures. Each deleted region is drawn as a red dotted line. The putative aa sequence in each mouse line is shown in black and red letters indicating WT and substituted aa residues, respectively. The gaps in the aa sequences are represented as red dashes. The characteristics of each group determined in this study are shown on the right. Mouse, embryonic lethality of homozygous mice. Protein, protein stability in the eight-week-old mouse cerebrum. mRNA, total expression level of the *Tardbp* mRNA. NE, not examined. Note that the putative protein structures of ZM93/Δ3 and ZM91/Δ1 mice (gray) are similar to those of ZM13A/Δ9 and ZE10/Δ10, respectively, and were not used in this analysis.

A long-disordered C-terminal region (CTR), known as a prion-like domain^2^, is also thought to be important for the stability of TDP-43 and its resistance to degradation. The CTR can be divided into four sub-regions, including a first Gly, aromatic, and Ser-rich (GaroS1) segment; a hydrophobic patch (HP); a Gln/Asn (Q/N)-rich segment; and a second GaroS2 segment (Fig. 1)^16,17^. In the CTR, an amyloidogenic core of amino acid (aa) residues 311–360 has been identified to be critical for aggregation^16^. In addition, the CTR is a hotspot for ALS-causing *TARDBP* mutations. These mutations accelerate aggregate formation, with the result that fragmented and phosphorylated C-termini of TDP-43 accumulate in the affected tissues of ALS patients^18–20^. Recently, TDP-ΔC (aa 274–414) knock-in mice were generated, and they showed embryonic lethality of homozygous mice, and that TDP-43 lacking CTR is unstable^21^. However, it is unclear which of the segments of the CTR contribute to the stability of TDP-43, indicating the importance of analyzing mice with deletions of more localized regions within the CTR. In this study, we generated 12 mouse lines lacking the various sub-regions of the CTR by genome editing and examined their effects on embryonic lethality of homozygotes, TDP-43 levels and subcellular localizations, and *Tardbp* mRNA levels reflecting autoregulation to clarify the functional diversity of the C-terminal sub-regions.

## Results

### Protein stability restoration is dependent on the length of the TDP-43 CTR

To determine the segment necessary to avoid embryonic lethality of TDP-ΔC knock-in mice^21^, we established 12 mouse lines using the zinc finger nuclease (ZFN) and CRISPR/Cas9 systems, which were used to target the Q/N-rich (N345–Q346) and GaroS2 (Y374) segments, respectively (Fig. 1, Supplementary Figs. S1, S2). The 12 mouse lines were categorized into five groups based on the putative protein structures in the CTR (Fig. 1): (1) deletion within a 6-aa region of the Q/N-rich segment (ZE13A/Δ9 and ZE13B/Δ18), (2) deletion within a 6-aa region of the GaroS2 segment (CRY9A/Δ3 and CRY8A/Δ18), (3) frame-shift deletions of the GaroS2 segment (CRY8B/Δ8, CRY9B/Δ29), (4) deletion of most of Q/N-GaroS2 (ZE10/Δ10, ZM39P/Δ295, and ZM87/Δ2), and (5) a large deletion including the GaroS1-HP segment (ZM39A/Δ261). All heterozygous mice were fertile and showed no obvious motor phenotypes during daily handling. Heterozygous intercrosses of each mouse line showed that homozygous mice were viable in Groups 1, 2, and 3, whereas they were embryonic lethal in Groups 4 and 5 (Table 1). Since embryonic lethality was recovered in Group 3 compared to Groups 4 and 5, the GaroS1-HP-Q/N-rich segment is thought to be necessary and sufficient for mouse development.

**Table 1:** Genotypes of offspring from heterozygous intercrosses. NM, natural mating. IVF, in vitro fertilization and embryo transfer. n/a, not applicable. *p < 0.05, **p < 0.01 (chi-square test).

Group	Mouse line	Mating	Stage	No. of mice with indicated genotype			No. of empty deciduae	No. of resorbed or dead fetuses
				+/+	+/Δ	Δ/Δ		
1	ZE13A/Δ9	NM	Pup	11	19	11	n/a	n/a
	ZE13B/Δ18	NM	Pup	11	20	5	n/a	n/a
2	CRY9A/Δ3	NM	Pup	13	27	12	n/a	n/a
	CRY8A/Δ18	NM	Pup	10	27	11	n/a	n/a
3	CRY8B/Δ8	NM	Pup	19	40	14	n/a	n/a
	CRY9B/Δ29	NM	Pup	19	39	15	n/a	n/a
4	ZE10/Δ10	NM	Pup	11	22	0**	n/a	n/a
		IVF	E12.5	10	20	0**	20	n/a
	ZM39P/Δ295	IVF	E12.5	7	11	0*	8	n/a
	ZM87/Δ2	NM	Pup	9	13	0*	n/a	n/a
		IVF	E19.5	10	22	0**	90	8 (Δ/Δ: 6)
		IVF	E16.5	2	11	4	11	2 (Δ/Δ: 2)
		IVF	E12.5	6	9	5	4	n/a
5	ZM39A/Δ261	NM	Pup	7	23	0**	n/a	n/a
		IVF	E12.5	12	28	0**	22	n/a

**Figure 2:**
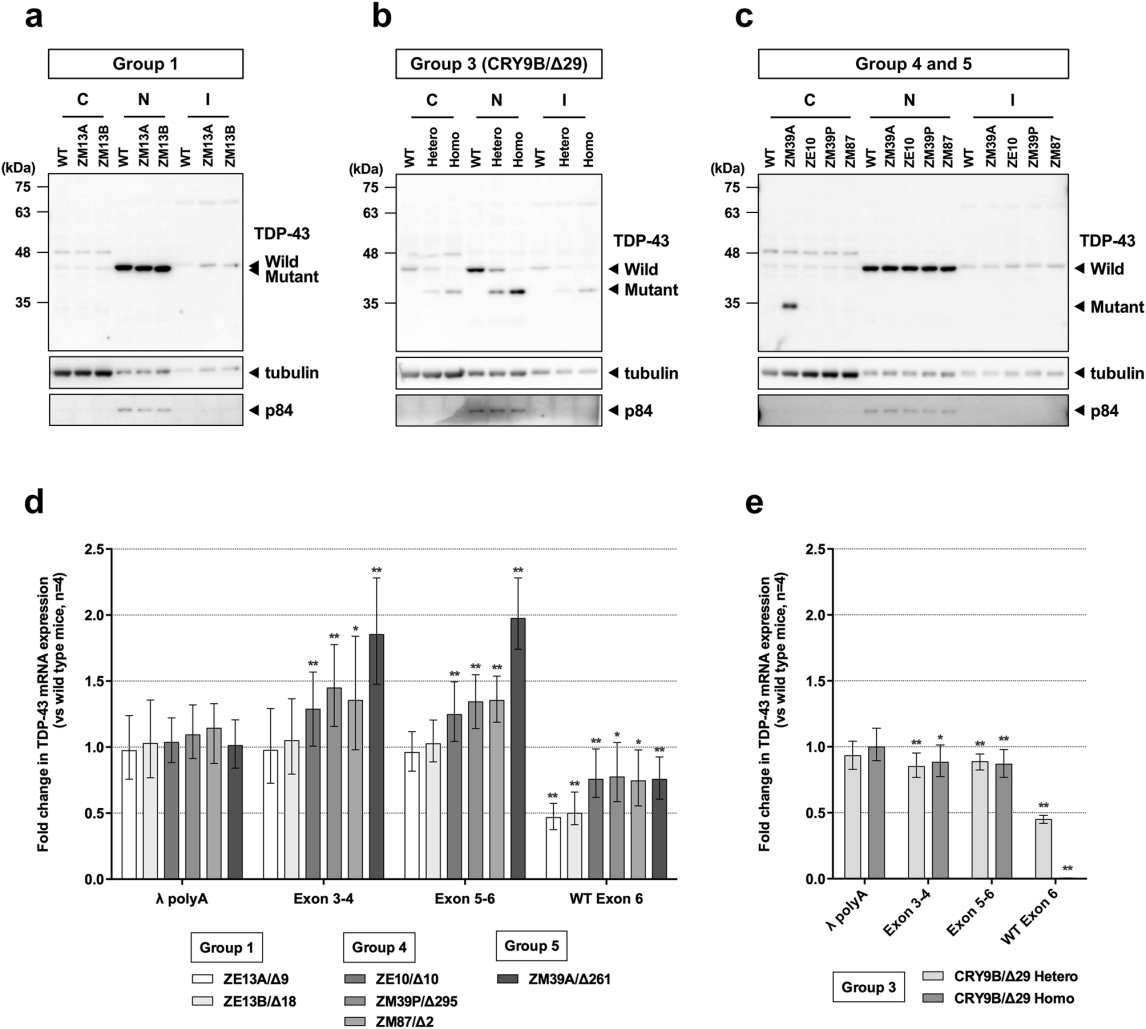
Western blotting and qRT-PCR analysis of the mice at eight weeks of age. (**a**–**c**) Cytoplasmic (C), nuclear (N), and insoluble (I) fractions of cerebrum samples from Group 1 homozygous mice (**a**), Group 3 heterozygous and homozygous mice (**b**), or Group 4 and 5 heterozygous mice (**c**) were subjected to western blotting using the antibodies indicated on the right. Anti-α-tubulin and anti-p84 antibodies were used as loading controls for the cytoplasmic and nuclear fractions, respectively. Full-length blots are presented in Supplementary Fig. S4. (**d**,**e**) Quantification of λpolyA (external control) and *Tardbp* mRNA levels normalized to *Hprt1* mRNA (internal reference). The total expression levels of *Tardbp* mRNA were evaluated using Exon 3–4 and Exon 5–6 primer sets. The WT Exon 6 primer set only recognized the WT allele. Each value indicates mean ± SEM as calculated by the REST2009 program. *p < 0.05, **p < 0.01.

To estimate the stability of mutant proteins, we performed subcellular fractionation of eight-week-old mouse cerebrum and western blot analysis using the N-260 anti-TDP-43 antibody. In Group 1 homozygous mice, mutant TDP-43 was observed mainly in the nucleus with a mobility shift consistent with a 3 or 6 aa deletion (Fig. 2a). In Group 3 heterozygous and homozygous mice, the intensity of the band corresponding to the GaroS2 deletion was comparable to that of wild-type (WT) TDP-43 depending on the number of alleles (Fig. 2b). However, mutant bands corresponding to most of the Q/N-GaroS2 deletion were not observable in the nuclear or cytoplasmic fractions of Group 4 heterozygous mice (Fig. 2c). Because protein stability was restored in Group 3 compared to Group 4, similar to embryonic lethality, the Q/N-rich segment is required for TDP-43 stability.

We noted that the timing of embryonic lethality was different in Group 4 mice (Table 1). The homozygous ZE10/Δ10 and ZM39P/Δ295 mice died before embryonic day (E) 12.5, similar to the previously described TDP-43 knock-out mice^4–6^, whereas ZM87/Δ2 mice died between E12.5 and E19.5, showing a variety of phenotypes including resorbed, macerated, anencephalic, or dead fetuses (Supplementary Fig. S3a). These results indicate that TDP-43 lacking most of Q/N-GaroS2 has residual function only in ZM87/Δ2 mice. Thus, we evaluated cerebral tissue from ZM87/Δ2 mice at E19.5 and detected weaker mutant bands both in heterozygotes and homozygotes compared to those of WT (Supplementary Fig. S3b). Furthermore, the ZM39P/Δ295 band was detected in unfertilized oocytes at an intensity that was comparable to those of WT and the ZM39A/Δ261 bands (Supplementary Fig. S3c), suggesting that TDP-43 lacking most of Q/N-GaroS2 is rapidly degraded in the cerebrum at eight weeks of age.

### Deletion of the GaroS2 segment induces a slight decrease in its own mRNA

Since TDP-43 strictly regulates its own mRNA levels through an autoregulatory negative feedback loop^9–13^, its residual function before being autoregulated in the nucleus, including nuclear TDP-43 level and activity, can be precisely detected by evaluating its own mRNA level. Thus, we performed qRT-PCR analysis using the following five primer sets: *Hprt1* (internal reference), λpolyA (external control), *Tardbp* exon 3 to 4 (Exon 3–4), *Tardbp* exon 5 to 6 (Exon 5–6), and *Tardbp* exon 6 (WT Exon 6). Exon 3–4 and Exon 5–6 recognize both WT and mutant alleles, whereas one of the WT Exon 6 primers targets deleted sequences induced by genome editing, thus WT Exon 6 recognizes the WT allele alone. In Group 1 homozygous mice, the total expression levels of *Tardbp* mRNA did not change in comparison with those of WT mice (Fig. 2d). However, the total expression levels were significantly increased in Groups 4 (1.247–1.448) and 5 (1.853–1.974), which were similar to TDP-ΔC knock-in mice^21^, indicating compensation for the loss of TDP-43 function. In sharp contrast, the total expression levels in both Group 3 heterozygotes and homozygotes were significantly decreased (0.850–0.887) compared to those of WT mice (Fig. 2e). These results suggest that the residual function of mutant TDP-43, which can induce downregulation of its own mRNA through autoregulation, is increased due to the deletion of the GaroS2 segment.

### CTR contributes to nuclear localization

**Figure 3:**
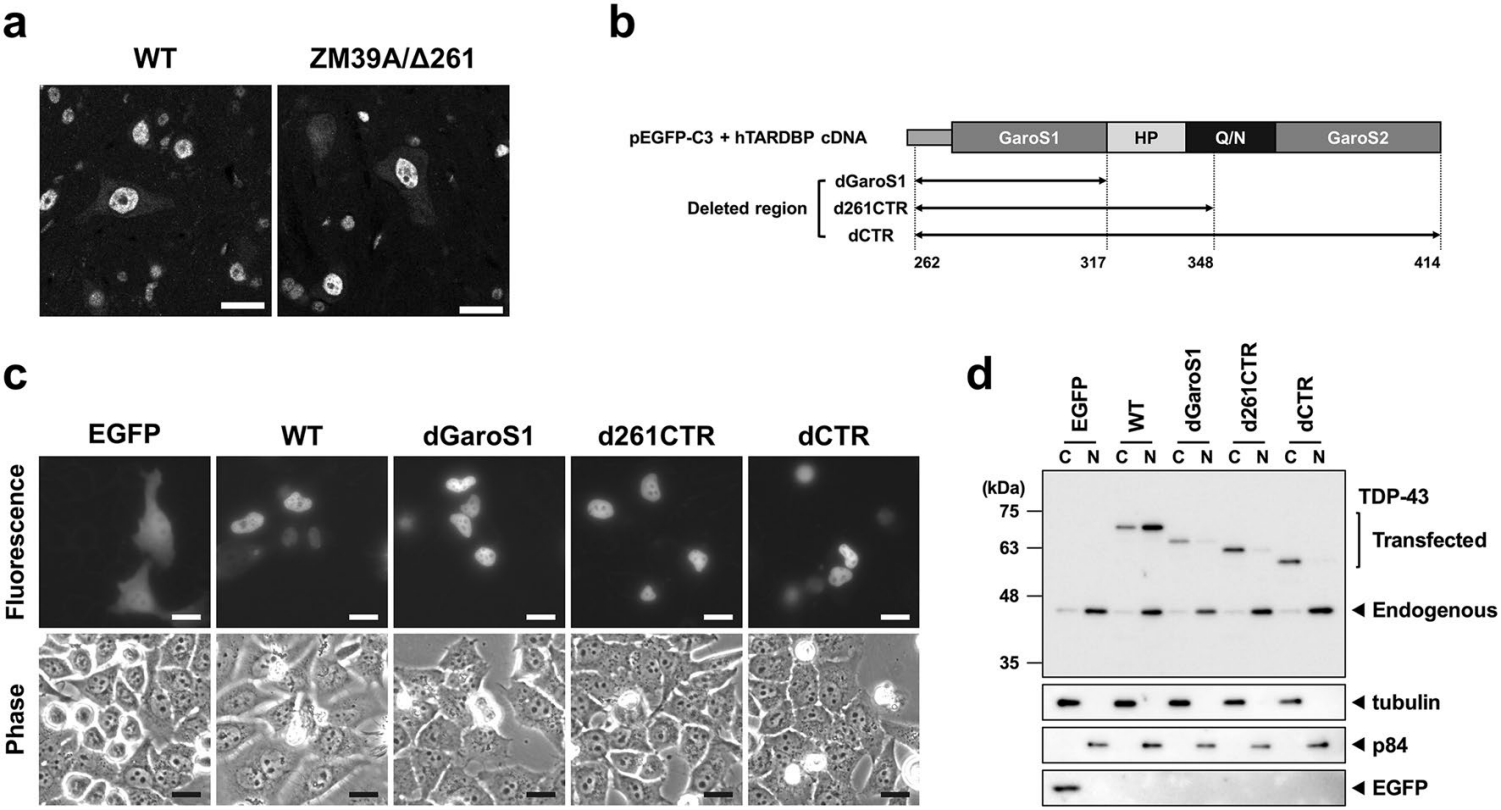
Subcellular localization of TDP-43 lacking various CTR deletions, including the GaroS1-HP segment. (**a**) Immunohistochemical analysis of the spinal anterior horn region in WT and ZM39A/Δ261 heterozygous mice stained with N-260 anti-TDP-43 antibody. Scale bar, 20 μm. (**b**–**d**) Transient expression of EGFP-tagged full-length human TDP-43 in HeLa cells. (**b**) Schematic diagram of full-length human TDP-43 constructs lacking dGaroS1 (aa 262–317), d261CTR (aa 262–346), or dCTR (aa 262–414). (**c**) Fluorescence and phase contrast images of living cells. Scale bar, 20 μm. (**d**) Cytoplasmic (C) and nuclear (N) fractions of cell lysates were subjected to western blotting using the antibodies indicated on the right. Note that the blot with the EGFP antibody shows only the low molecular weight region (approximately 27 kDa). Full-length blots are presented in Supplementary Fig. S4.

As shown in Fig. 2c, Group 5 heterozygous mice (ZM39A/Δ261) exhibited a strong signal for loss-of-function TDP-43 lacking the GaroS1-HP segment, which was detected in the cytoplasmic fraction. Although the loss-of-function TDP-43 had a bipartite nuclear localization signal (NLS, aa 82–98, Fig. 1)^22,23^, it was not observed in the nuclear fraction. We performed immunohistochemical analysis of the spinal anterior horn region stained with N-260 anti-TDP-43 antibody, but the mis-localization of mutant TDP-43 was not detected (Fig. 3a). To confirm whether a similar result occurs in human TDP-43, we performed transient expression analysis using HeLa cells with enhanced green fluorescent protein (EGFP) tagged to the N-terminus of full-length human TDP-43 constructs lacking the GaroS1 segment (dGaroS1), a region equivalent to ZM39A/Δ261 (d261CTR) and the entire CTR (dCTR) (Fig. 3b). Similar to the mouse immunohistochemical analysis, three mutant and WT TDP-43s were localized in the nuclei of living cells, whereas EGFP alone was diffusely localized in cell bodies (Fig. 3c). In contrast to the fluorescence imaging, subcellular fractionation followed by western blotting showed that the three mutant TDP-43s were strongly detected in the cytoplasmic fraction (Fig. 3d). Although the reason is unclear, a similar discrepancy has been reported using constructs with a near total deletion of CTR (aa 314–414)^23,24^. Taking these reports into account, large deletions of CTR appear to disturb the nuclear localization only when assessed by subcellular fractionation and western blotting.

## Discussion

We established 12 mouse lines lacking various TDP-43 C-terminal sub-regions and demonstrated that extending the length of CTR to the GaroS1-HP-Q/N-rich segment restored the protein stability of TDP-43. On the other hand, loss-of-function TDP-43 lacking the GaroS1-HP segment was stably expressed and detected in the cytoplasmic fraction, indicating that the stability of TDP-43 does not just depend on its residual function. The TDP-ΔC protein is known to be unstable in knock-in mice^21^. Moreover, mammalian TDP-43 genes undergo alternative splicing to produce TDP-43 lacking most of the CTR, namely mTDP-S^25^. Our results strongly suggest that mTDP-Ss are rapidly degraded in the cerebrum. In addition to protein stability, we found that the GaroS2 segment is not essential for mouse development.

The protein structure in solution exhibits two canonical α-helices (321–330 and 335–343) in the HP segment^16^, which overlaps a highly conserved region (CR, 320–340) flanked by two intrinsically disordered regions (IDR1 and IDR2)^26^. Hydrophobic residues (V, L, I, M, F, Y, and W) in the IDRs are distributed at conserved intervals across vertebrate TDP-43 homologues. These regions are cooperatively involved in liquid–liquid phase separation (LLPS) and forming membraneless organelles^27–29^. The self-association of CTR is synergistic and forms a continuum of physical states, which is proposed to be mainly facilitated by four interacting regions in the GaroS1-HP-Q/N-rich segment, α-helices (aa 321–330), a low complexity aromatic-rich kinked segment (LARKS, aa 312–317), a steric zipper (aa 328–333), and a fibril core (aa 331–360)^30,31^. In contrast, the GaroS2 segment is not necessary for full-length TDP-43 aggregation^32^. These observations suggest that the basic elements necessary for functional LLPS are the GaroS1-HP-Q/N-rich segment.

It has been shown that removing the GaroS2 segment has no apparent effect on either the TDP-43 activity of CFTR exon 9 skipping or intra-cellular localization in cultured cells^23,33^. These reports support our results showing no apparent phenotype in CRY9B/Δ29 mice lacking the GaroS2 segment. However, there are several mutations in the GaroS2 segment that cause familial ALS, and the Y374X mutation, similar to the CRY9B/Δ29 mutation, has been found in sporadic ALS^34^. Furthermore, two steric zippers (aa 370–375 and 396–402) are present in the GaroS2 segment^30^. It is noteworthy that the GaroS2 segment is subjected to phosphorylation at a minimum of seven serine residues (aa 389, 393, 395, 403, 404, 409, and 410)^18–20^, which may serve to prevent excessive aggregation^31^. Considering that disordered proteins are generally regulated by post-translational modifications (PTMs)^35^, it may be that the GaroS2 segment is not essential for basic TDP-43 activity, but rather regulates its own nuclear function including TDP-43 activity and stability, presumably due to PTMs including phosphorylation.

We used western blotting to show that full-length human TDP-43 with larger CTR deletions localized mainly in the cytoplasmic fraction, whereas the protein constructs were present in the nuclei of living cells when imaged using fluorescence microscopy. In a pioneering study using U2OS cells and CTR deletion constructs, the observed immunofluorescence patterns of subcellular localizations were variable, whereas biochemical fractionation showed that cytoplasmic localization was dependent on the length of the CTR deletion^23^. Recently, TDP-43 was reported to demix in the cytoplasm as well as the nucleus using CTR^36^. The demixing of RNA-free TDP-43 in the nucleus induces anisotropic intranuclear liquid spherical shells (anisosomes), and the role of the demixing is speculated to tether RNA binding proteins, including TDP-43, near the site of transcription^37^. Furthermore, a nuclear export signal (aa 239–250) in TDP-43 appears to be non-functional, and a significant amount of TDP-43 is exported to cytoplasm through passive diffusion^38,39^. These observations raise the possibility that TDP-43s with large CTR deletions, including TDP-43 lacking the GaroS1-HP segment in Group 5 mice, can be imported to the nucleus, but they are dysfunctional and leak out during biochemical fractionation due to insufficient nuclear retention. Although further analysis and mouse models are required to understand the full functional diversity of the TDP-43 C-terminal sub-regions, we expect that the results presented here will help elucidate the pathogenesis of ALS and perhaps lead to the development of new ALS treatments.

## Methods

### Generation of ZE, ZM, and CRY mouse lines

CompoZr Custom ZFNs (Sigma-Aldrich, St. Louis, MO, USA) were designed to target *Tardbp* exon 6 and manufactured as ZFN binding (uppercase) and cutting (lowercase) sites (GTTAGCCAGCCAGCAGaaccagTCGGGCCCATCTGGGA). The ZFN mRNAs were microinjected into the pronuclei of B6C3F1/Jcl fertilized oocytes to generate ZE lines according to the manufacturer’s instructions. Next, a ssDNA oligonucleotide (Supplementary Fig. S1a) was co-injected with the ZFN mRNAs as a donor template to generate the ZM lines carrying large deletions.

To generate GaroS2-deficient mice, the guide RNA (gRNA) target sequence (TCTGGAAATAATTCCTACAGtgg, lowercase indicating protospacer adaptor motif, PAM) was designed to target *Tardbp* exon 6 encoding the N-terminal region of the GaroS2 segment. The gRNA was synthesized commercially (Thermo Fisher Scientific, Waltham, MA, USA). The gRNA and CAS9 protein (Integrated DNA Technologies, Coralville, IA, USA) were electroporated in fertilized zygotes of C57BL/6JJcl mice using previously described methods with minor modifications^40^. Electroporation was performed in HEPES-buffered Whitten’s medium using platinum plate electrodes on tempered glass (LF501PT-1; BEX, Tokyo, Japan). The pulse conditions of CUY21EDIT II (BEX) were 30 V (3 ms pulse duration, 97 ms interval) × 7 times. After electroporation, zygotes were incubated in Whitten’s medium for 24 h at 37 °C and 5% CO_2_, and the surviving two-cell-stage embryos were transferred into the oviducts of pseudo-pregnant female mice.

The genome-edited founder mice were crossed with C57BL/6JJcl mice to obtain heterozygous mouse lines. The genotyping conditions are described in Supplementary Table S1. All animal experiments were carried out in accordance with the guidelines of the National Institutes of Health, and the Ministry of Education, Culture, Sports, Science and Technology (MEXT) of Japan, and were approved by the Dean of Kitasato University School of Medicine based on judgment by the Institutional Animal Care and Use Committee (Approval no. 2020-061) in compliance with ARRIVE guidelines (https://arriveguidelines.org). All mice were maintained under specific pathogen-free conditions with free access to CE-2 standard food (CLEA, Tokyo, Japan) and ultrafiltered water. For the mouse lines where embryonic lethality was expected, including ZM39P/Δ295, the analysis at E12.5 was prioritized to reduce the number of mice.

### Subcellular fractionation and western blotting

Cerebrum samples from eight-week-old mice were homogenized and divided into four subcellular fractions enriched with cytoplasmic (C), membrane (M), nuclear (N), or insoluble (I) proteins using EzSubcell Extract (WSE-7421, ATTO, Tokyo, Japan). The fractions (C, N, and I), 3 µg per lane, were subjected to western blotting as described previously^41^. The blots were subsequently incubated with the following antibodies: TDP-43 (1:2000, 10782-2-AP, Proteintech, Chicago, IL, USA), α-tubulin (1:5000, T5168, Sigma-Aldrich), or p84 (1:1000, ab131268, Abcam, Cambridge, UK). For subcellular fractionation of cultured cells, the Pierce NE-PER reagents (Thermo Fisher Scientific) were applied according to the method used by Nonaka et al.^24^, and antibodies for p84 (1:1000, GTX70220, GeneTex, Irvine, CA, USA) and GFP (1:1000, sc-9996, Santa Cruz Biotechnology, Dallas, TX, USA) were used for detection.

### Total RNA preparation and qRT-PCR analysis

To monitor the measurement process, we spiked external RNA (λpolyA^+^ RNA-A) from an External Standard Kit for qPCR (Takara, Shiga, Japan) into the cerebral homogenate at a concentration of 1.8 × 10^6^ copies/mg brain tissue, and simultaneously extracted total RNA from the cerebrum using RNAiso Plus (Takara). First-strand cDNA was synthesized using a SuperScript VILO cDNA synthesis Kit (Invitrogen, Waltham, MA, USA). Real-time qRT-PCR was performed using SYBR Premix Ex Taq II and a Thermal Cycler Dice Real-time System (Takara). Each cDNA sample was measured in duplicate applying the ΔΔCT method. Statistical analyses were performed using the REST2009 program (http://www.gene-quantification.de/rest-2009.html). The primer sets are listed in Supplementary Table S1.

### Immunohistochemistry

Immunohistochemical analysis was performed as described previously^42^. Paraffin-embedded 3-µm-thick sections were subjected to immunostaining with N-260 anti-TDP-43 antibody (1:5000, 10782-2-AP, Proteintech). Immunofluorescence images were obtained using Alexa Fluor 488 goat anti-rabbit IgG (1:2000, A11034, Invitrogen) and a LSM710 confocal laser microscope (Carl Zeiss, Jena, Germany).

### Cell culture and fluorescence imaging

WT human TDP-43 cloned into the pEGFP-C3 vector (Clontech, now Takara) was constructed previously^43^. Deletion constructs were generated with a PrimeSTAR Mutagenesis Basal Kit (Takara) using the following primers: for dGaroS1, 5′-TGCCGAAATTAATCCAGCCATGATG-3′ and 5′-GGATTAATTTCGGCATTGGATATATG-3′; for d261CTR, 5′-TGCCGAACCATCGGGTAATAACCAA-3′ and 5′-CCCGATGGTTCGGCATTGGATATATG-3′. The dCTR construct was obtained from multicopy insertions of primer oligonucleotides during d261CTR construction. These deletion constructs were purified with an EndoFree Plasmid Maxi kit (Qiagen, Hilden, Germany) and transfected in HeLa cells using Polyethylenimine MAX (PEI “MAX”, Polysciences, Warrington, PA, USA). After transfection, HeLa cells were cultured in DMEM supplemented with 10% fetal bovine serum in 5% CO_2_ at 37 °C for 48 h. The cells were then harvested for subcellular fractionation or subjected to fluorescence imaging using an inverted microscope, Axio Vert.A1FL-LED (Carl Zeiss).

## Supplementary Information

Supplementary Information.
