# Supplementary material for: Importance of the Q/N-rich segment for protein stability of endogenous mouse TDP-43
Source: Sci Rep. 2022 Sep 2;12:14923. doi: 10.1038/s41598-022-19153-0 (PMC9440050; doi:10.1038/s41598-022-19153-0)
Supplement: Supplementary file 1 — Supplementary Information. [file 41598_2022_19153_MOESM1_ESM.docx]

**Electronic supplementary material**

**Importance of the Q/N-rich segment for protein stability of endogenous mouse TDP-43.**

Toshiya Sato^1,2,3^, Kanako Oda^3^, Seiko Sakai^3^, Rika Kato^2^, Saori Yamamori^4^, Makoto Itakura^4^, Yoshio Kodera^5^, Masatoyo Nishizawa^6,7^, Toshikuni Sasaoka^3^, Osamu Onodera^6^, Minesuke Yokoyama^3,8^

^1^Department of Laboratory Animal Science, Kitasato University School of Medicine, Sagamihara 252-0374, Japan.

^2^Center for Genetic Studies of Integrated Biological Functions, Kitasato University School of Medicine, Sagamihara 252-0374, Japan.

^3^Department of Comparative and Experimental Medicine, Brain Research Institute, Niigata University, Niigata 951-8585, Japan.

^4^Department of Biochemistry, Kitasato University School of Medicine, Sagamihara 252-0374, Japan.

^5^Department of Physics, Kitasato University School of Science, Sagamihara 252-0373, Japan.

^6^Department of Neurology, Brain Research Institute, Niigata University, Niigata 951-8585, Japan.

^7^Department of Nursing, Niigata University of Health and Welfare, Niigata 950-3198, Japan.

^8^Central Institute for Experimental Animals, Kawasaki 210-0821, Japan.

**
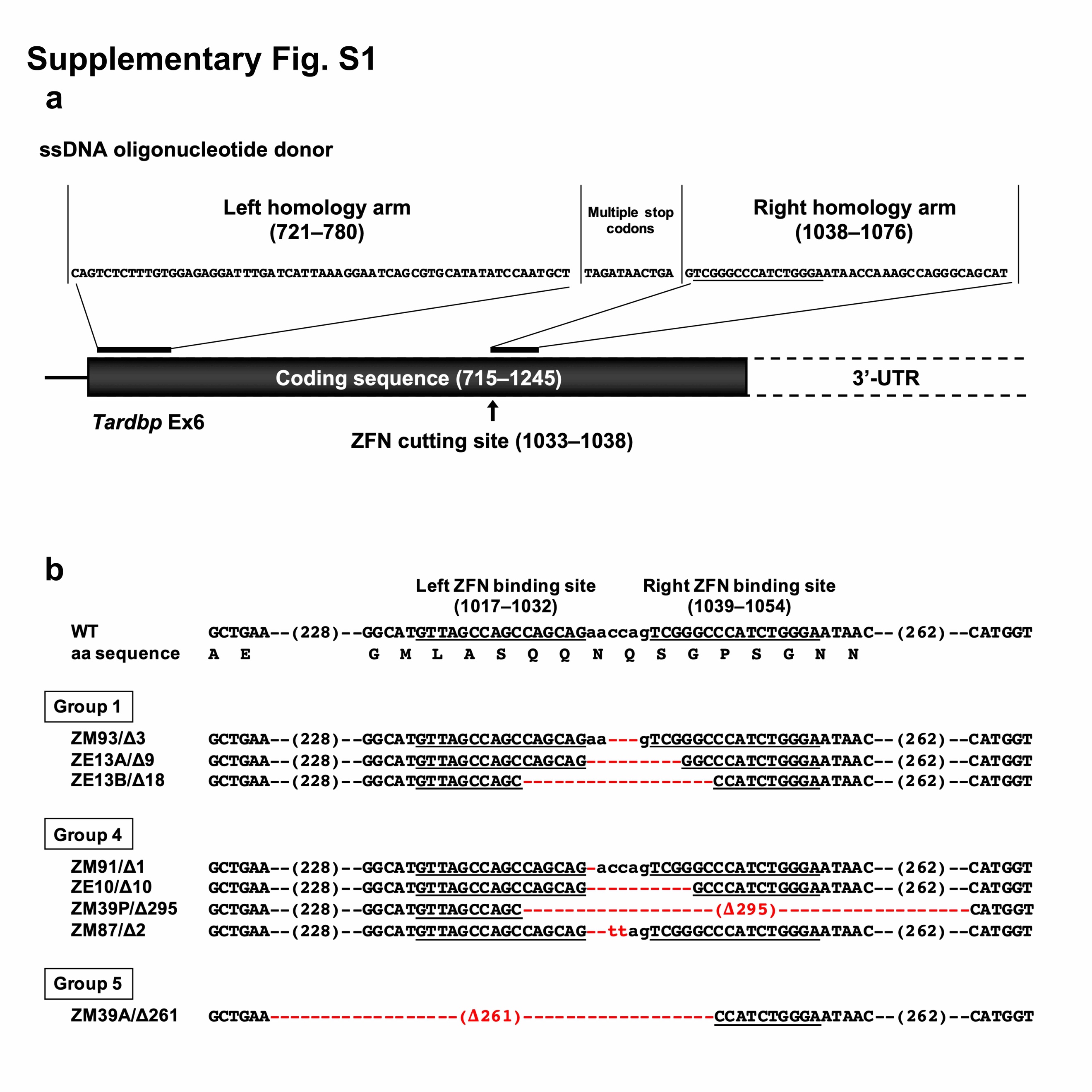
**

**Fig. S1.** Generation of ZE and ZM mouse lines. (**a**) Schematic diagram of *Tardbp* exon 6 and the ssDNA oligonucleotide co-injected with the ZFN mRNAs as a donor template. The base number of the *Tardbp* coding sequence is given in parentheses. (**b**) The nucleotide sequences of the mutant alleles in the ZE and ZM mouse lines. We obtained six founders, including mosaic mice, and separated them into eight mouse lines by mating with C57BL/6 mice. ZFN-mediated nucleotide deletions of 1 to 295 bp were observed around the ZFN target. The nucleotide deletions and substitutions are represented as red dashes and letters, respectively. ZFN binding and cutting sites are indicated by underlining and lowercase letters, respectively. Note that multiple stop codons in the ssDNA oligonucleotide were deleted in the ZM mouse lines. Thus, the resulting ZM lines have only large (ZM39A/Δ261 and ZM39P/Δ295) or small (ZM87/Δ2 and ZM91/Δ1) deletions.

**
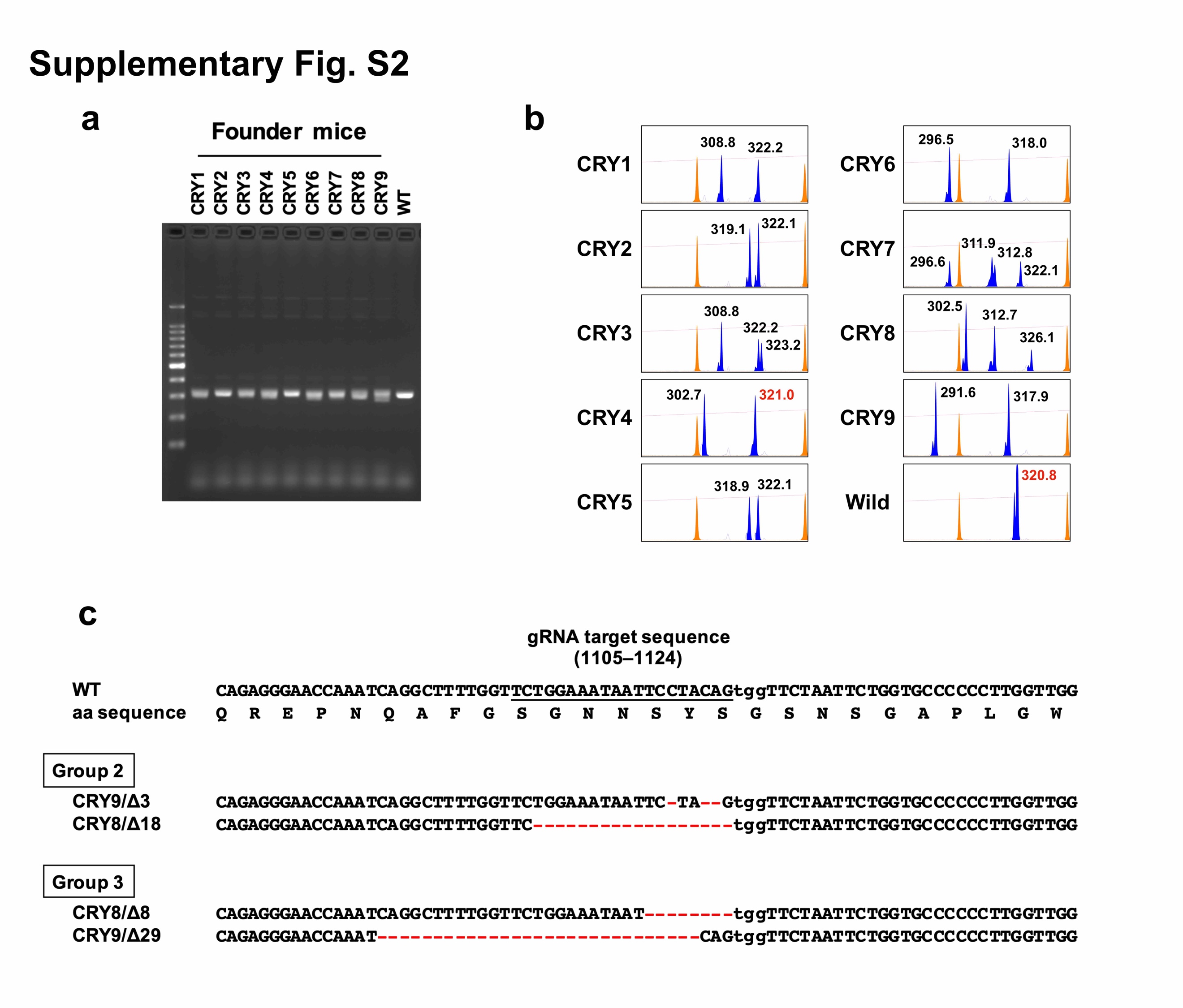
**

**Fig. S2.** Generation of CRY mouse lines. (**a** and **b**) Genomic PCR products of seven female and two male founder mice (CRY1–9) were electrophoresed using 2% agarose gel (**a**) and an ABI 3130 xL DNA sequencer (Applied Biosystems, Foster City, CA) (**b**). The sizes of the PCR products (blue chromatogram) were calculated by Peak Scanner Software v1.0 (Applied Biosystems) using a GeneScan 500 LIZ dye Size Standard (orange chromatogram) and are shown in black (21 mutant alleles) and red (same size as wild type) numbers. (**c**) The nucleotide sequences of four mutant alleles established from two male founder mice (CRY8–9). CRISPR/Cas9-mediated nucleotide deletions of 3 to 29 bp were observed around the gRNA target. The nucleotide deletions are represented as red dashes. The gRNA target sequence and PAM are indicated by underlining and lowercase letters, respectively.

**
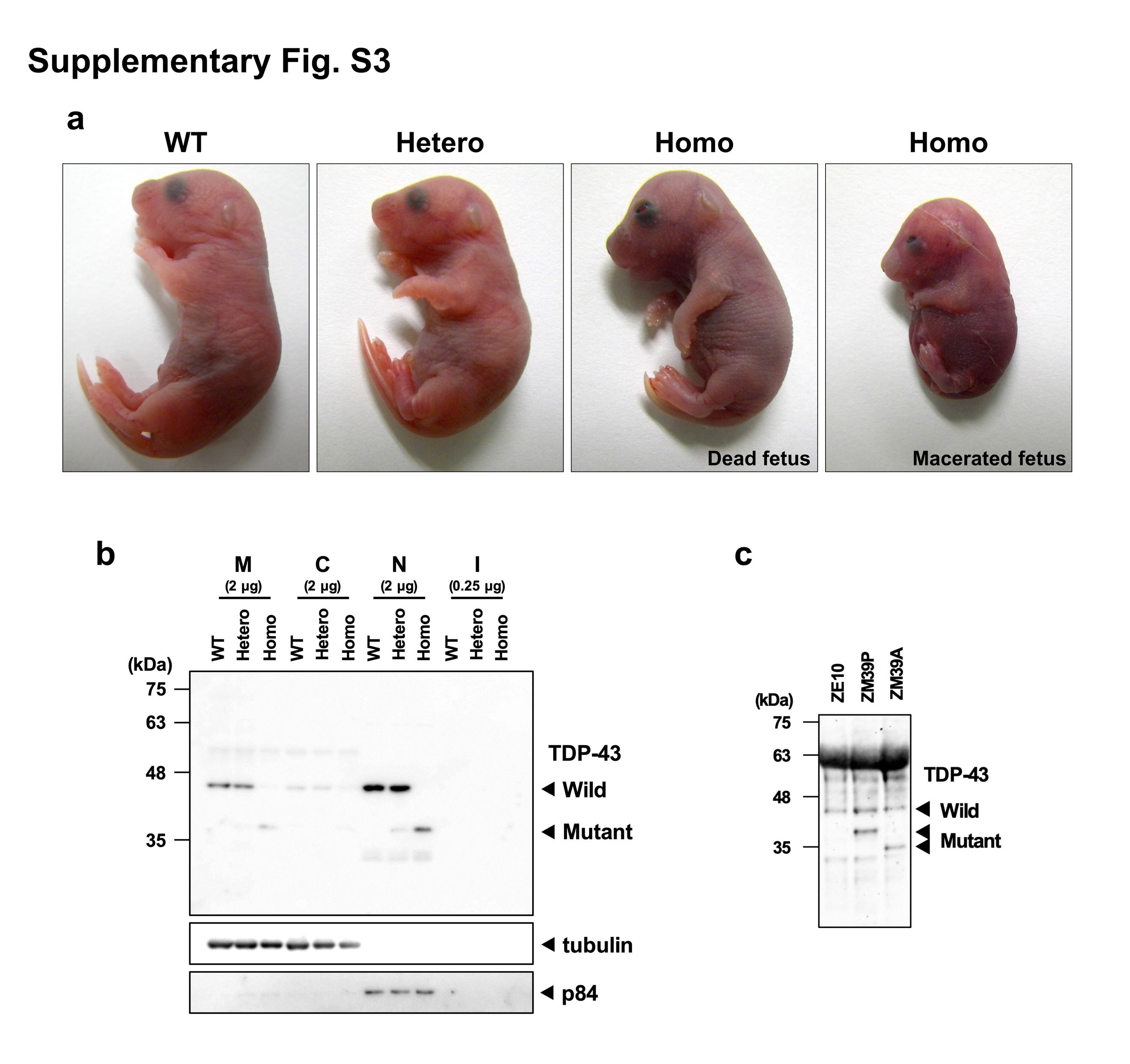
**

**Fig. S3.** Developmental analysis of TDP-43 mutant mice. (**a**) Representative photographs of ZM87/Δ2 fetuses at E19.5 obtained by *in vitro* fertilization and embryo transfer from heterozygous intercrosses followed by cesarean section. In some cases, ZM87/Δ2 homozygous fetuses grew to approximately the same size as the wild type (Dead fetus, third panel from left), but were edematous and did not start breathing. (**b**) Western blotting of ZM87/Δ2 mice at E19.5. Membrane (M), cytoplasmic (C), nuclear (N), and insoluble (I) fractions of each cerebrum were subjected to western blotting using the antibodies indicated on the right. Note that the protein in the insoluble fraction was reduced because of poor yield. (**c**) Western blotting of unfertilized oocytes obtained by superovulation of ZE10/Δ10, ZM39P/Δ295, and ZM39A/Δ261 female mice. To estimate the TDP-43 as maternal products, 10 unfertilized oocytes were directly dissolved in sample buffer and subjected to western blotting.


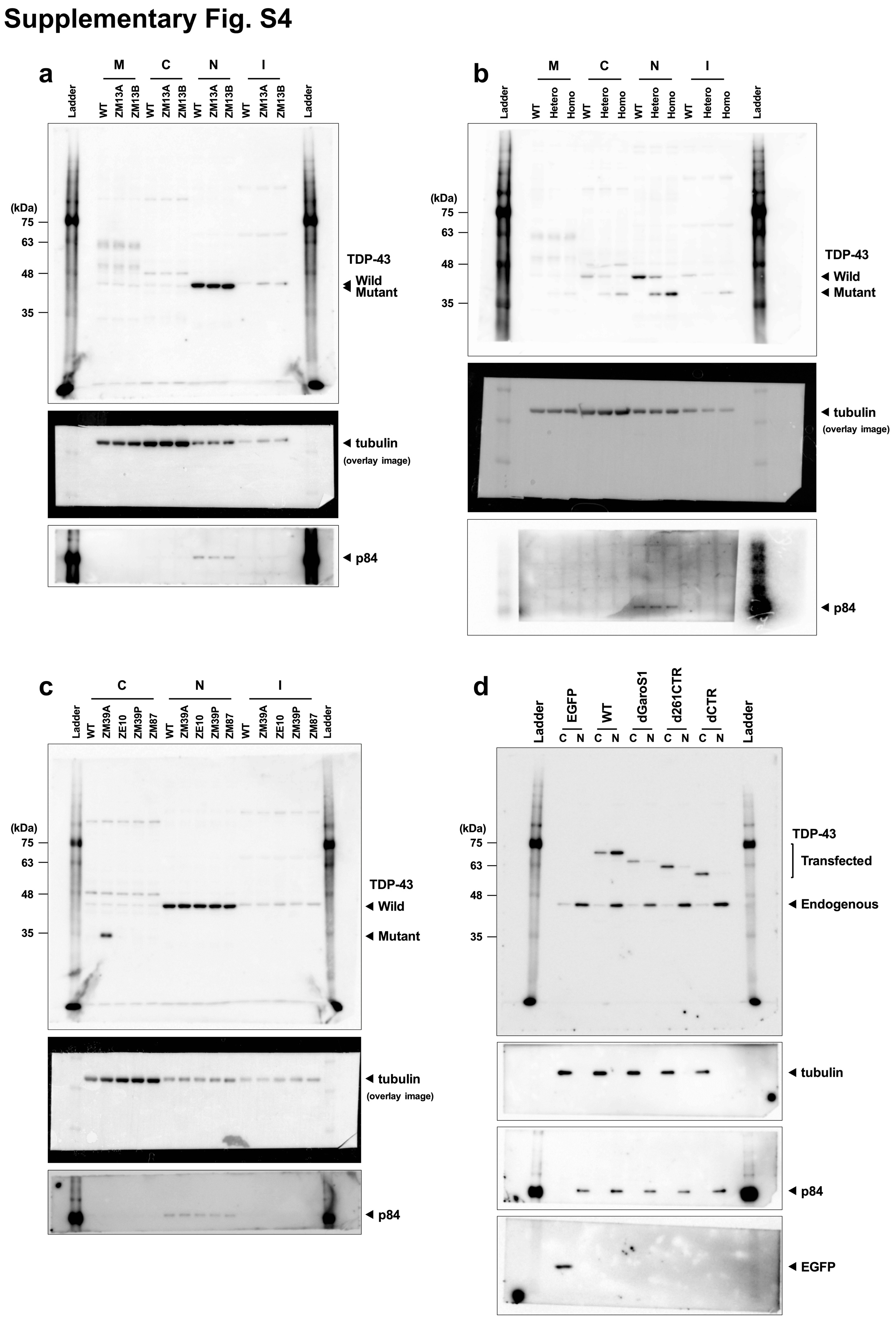


**Fig. S4.** Full-length blots of Figs 2a (**a**), 2b (**b**), 2c (**c**), and 3d (**d**). After staining with N-260 anti-TDP-43 antibody, the antibody was stripped, the blotted membranes were trimmed to the expected molecular weight range, and each trimmed membrane was re-probed with anti-α-tubulin, anti-p84, or anti-GFP antibody. In the anti-α-tubulin blots (**a**, **b,** and **c**), membrane edges were hardly visible due to the high signal-to-noise ratio. Therefore, the overlay images of white light and chemiluminescence imaging were provided to show the full-length blots. Note that the protein ladders on both sides of the anti-p84 blot (**b**) were hidden with paper.

**Table S1. PCR primers and conditions**

| **Primers for fragment analysis** | | | |
| --- | --- | --- | --- |
| **Target** | **Primer** | **Genotyping** | **PCR condition** |
| Exon 6 | CAGGGTGGGTTTGGTAACAG | All mice except for ZM39 mice | 95°C 30 s, 55°C 30 s, 72°C 30 s, 35 cycles |
|  | FAM-GATCCTGCATTTGATGCTGA |  | TaKaRa Ex Taq (Takara) |
| **Primers for conventional PCR** | | | |
| **Target** | **Primer** | **Genotyping** | **PCR condition** |
| Intron 5 | ACTGCTGTGTTGATATCTGGGTTG | ZM39A/Δ261 | 94°C 15 s, 62°C 15 s, 72°C 30 s, 35 cycles |
| Exon 6 | TGCCCTGGCTTTGGTTATTCC |  | KAPATaq Extra (Kapa Biosystems) |
| Exon 6 | CAGCGTTGCAGAGCAGTTG | ZM39P/Δ295 | 94°C 15 s, 62°C 15 s, 72°C 30 s, 35 cycles |
|  | CAGCAGTTCACTTTCACCCACTC |  | KAPATaq Extra (Kapa Biosystems) |
| Exon 6 | CAGCGTTGCAGAGCAGTTG | CRY9B/Δ29  CRY8A/Δ18 | 94°C 15 s, 71-67°C (-0.5/cycle) 10 s, 72°C 15 s, 30 cycles |
|  | ATTAAAACCACTGCCCGATCCT |  | KAPATaq Extra (Kapa Biosystems) |
| **Primers for qRT-PCR** | | | |
| **Target** | **Primer** | **Application** | **PCR condition** |
| Exon 3 | CTCCCCTGGAAAACAACTGAGC | All mice | 95°C 5 s, 60°C 30 s, 40 cycles |
| Exon 4 | ATCGAACAAAGCCAAACCCTTTC |  | SYBR Premix Ex Taq II (Takara) |
| Exon 5 | AGAGGACATGACTGCTGAAGAGC | All mice | 95°C 5 s, 60°C 30 s, 40 cycles |
| Exon 6 | CTCCACAAAGAGACTGGGCAAC |  | SYBR Premix Ex Taq II (Takara) |
| Exon 6 | TAGCCAGCCAGCAGAACCAGT | ZE and ZM lines | 95°C 5 s, 60°C 30 s, 40 cycles |
|  | GGGGCACCAGAATTAGAACCAC |  | SYBR Premix Ex Taq II (Takara) |
| Exon 6 | TAGCCAGCCAGCAGAACCAGT | CRY lines except for CRY9A/Δ3 | 95°C 5 s, 60°C 30 s, 40 cycles |
|  | GCACCAGAATTAGAACCACTGTAGG |  | SYBR Premix Ex Taq II (Takara) |
| Hprt1 | Real Time Primer MA031262 (Takara) | All mice | 95°C 5 s, 60°C 30 s, 40 cycles |
|  |  |  | SYBR Premix Ex Taq II (Takara) |
| λpolyA | Real Time Primer for λpolyA #3786  (Takara) | External control | 95°C 5 s, 60°C 30 s, 40 cycles |
|  |  |  | SYBR Premix Ex Taq II (Takara) |

Primer sequences are 5’ to 3’.
